# A novel heterozygous mutation in ANK1 solves a mystery of a patient with hyperbilirubinemia and splenomegaly

**DOI:** 10.12669/pjms.42.4.12871

**Published:** 2026-04

**Authors:** Yixian Shi, Yang Ou, Haicong Wu

**Affiliations:** 1 Yixian Shi Department of Surgical Intensive Care Unit, Mengchao Hepatobiliary Hospital, Fujian Medical University, Fuzhou 350007, Fujian, China; 2 Yang Ou Department of Surgical Intensive Care Unit, Mengchao Hepatobiliary Hospital, Fujian Medical University, Fuzhou 350007, Fujian, China; 3 Haicong Wu, Liver Research Center of the First Affiliated Hospital of Fujian Medical University, Fuzhou 350001, Fujian, Chin

**Keywords:** ANK1, Case report, Hyperbilirubinemia, Hereditary spherocytosis, Splenomegaly

## Abstract

Patients presented with jaundice and splenomegaly are easily diagnosed as having liver diseases. Actually, some atypical cases belong to hemolytic diseases leading to secondary hemochromatosis, which is easily misdiagnosed or never diagnosed. Here, we describe an atypical case in a 27 years old man presenting with unconjugated hyperbilirubinemia and splenomegaly since early childhood, who was hospitalized to check for any possible causes related to liver diseases. However, the probable etiology related to hemolytic diseases and liver disease was ruled out by negative traditional tests, which left a mystery to us. Fortunately, next-generation sequencing is becoming a suitable choice to determine the candidate genes responsible for rare or inherited disorders. A novel de novo mutation (c.5032delA) was firstly identified through whole exome sequencing which could induce an arginine to glycine substitution at residue 1678 (p.R1678Gfs*12), causing a premature termination codon in exon 38 of ANK1. ANK1 is involved in erythrocyte cytoskeleton formation, and contributes to one of the most common causes of hereditary spherocytosis (HS). Besides, homology-modeling analysis confirmed the loss-of-function of frameshift variant of ANK1 with bioinformatics methods. This work adds new knowledge in the etiology of hereditary spherocytosis. In addition, genetic testing can open up new perspectives for atypical and unknown diseases when traditional tests cannot be met.

## INTRODUCTION

Patients presented with jaundice and splenomegaly are easily diagnosed as having liver diseases. Actually, some atypical cases belong to hemolytic diseases leading to secondary hemochromatosis, which is easily misdiagnosed or never diagnosed. Here, we describe an atypical case of a young man presenting with unconjugated hyperbilirubinemia, splenomegaly and anisocytosis, which was confusing. Eventually, he was found to have hereditary spherocytosis (HS) identified by next-generation sequencing (NGS), which was confirmed validated by predicted three-dimensional (3D) structure of mutant ANK1 through the I-TASSER server.

## CASE PRESENTATION

**Fig.1:**
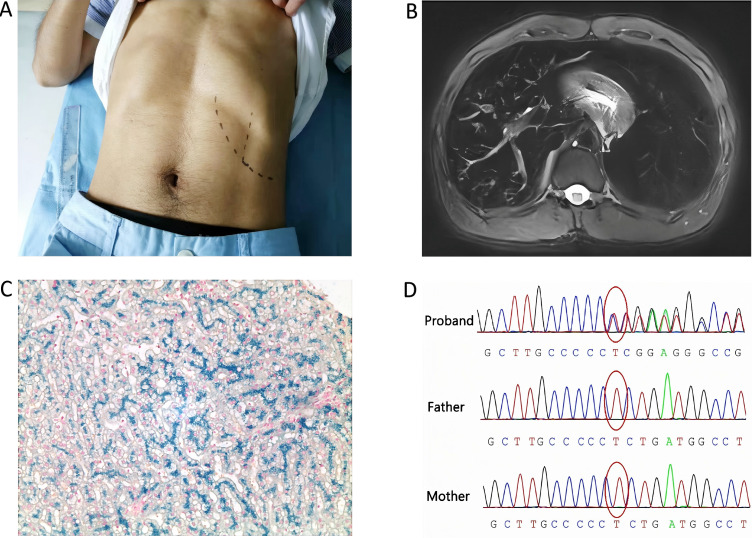
Clinical manifestations of the patient. A: Physical examination disclosed a palpable spleen; B: Abdominal MRI revealed marked splenomegaly and diffuse low signal intensity of liver and spleen in T2WI; C: Hemosiderin deposition in liver biopsy (Prussian blue iron staining, ×100); D: Sanger sequencing validation of the ANK1 c.5032delA mutation. DNA was extracted from peripheral blood mononuclear cells from the proband and his family members.

A 27 years old man with hyperbilirubinemia and splenomegaly since early childhood was hospitalized for determination of their etiology. Written informed consent was obtained from the participant for the publication of this case report and any accompanying clinical data. The patient had no previous medical history. Neither of the patient’s parents had a history of jaundice, splenomegaly, or anemia. In addition, the patient had no history of gallstones or blood transfusion. Physical examination disclosed jaundice and a palpable spleen (7 cm below the costal margin) (Fig.1A). No hepatomegaly was detected on admission. He had no anemic face, liver palm or spider mole. Liver tests revealed alanine aminotransferase 10 U/L, aspartate aminotransferase 19 U/L, g-glutamyl transferase 14 U/L, alkaline phosphatase 59 U/L, total bilirubin 116.3 μmol/L (unconjugated fraction 108.1 μmol/L) and an international normalized ratio of 1.25. Routine blood examination showed: hemoglobin 132 g/L, platelet count 215×10^9^/L, and reticulocytes 12.96%. Peripheral red blood cells varied in size and shape. Glucose-6 phosphate dehydrogenase activity, osmotic fragility, direct Coombs’ test, Ham’s test and sucrose lysis test were all negative. Hemoglobin electrophoresis showed no abnormality and a genetic variant analysis for α- and β-thalassemia was negative. Viral hepatitis (A-E) was ruled out. Ceruloplasmin and autoimmune markers were normal. Abdominal magnetic resonance imaging (MRI) revealed marked splenomegaly and diffuse low signal intensity of liver and spleen in T2-weighted imaging (T2WI), suggesting hemosiderin deposition (Fig.1B). The bone marrow punct ure and liver biopsy revealed erythroid hyperplasia and hepatic hemosiderosis (Fig.1C). Moreover, hereditary unconjugated hyperbilirubinemia-Gilbert syndrome was excluded because nonsense mutations were detected in UGT1A1 gene.

As mentioned above, the probable etiology related to hemolytic diseases and liver disease was ruled out by negative traditional tests, which left a mystery to us. Fortunately, next-generation sequencing is becoming a wise choice to determine the candidate genes responsible for rare or inherited disorders. Whole exome sequencing (WES) was finally performed by Running Gene Inc. (Beijing, China) to discover the causal gene according to the manufacturer’s instructions.^1^ A novel heterozygous mutation c.5032delA in exon 38 of ANK1 was identified and confirmed by Sanger sequencing (Fig.1D). The frameshift variant, located in the regulatory domain in the C terminus of ANK1, induced an arginine to glycine substitution at residue 1678 (R1678Gfs*12), leading to truncation of the ankyrin1 (ANK1) protein. His parents did not carry this mutation. Thus, the proband had a de novo mutation in ANK1. This mutation was never previously demonstrated in the HGMD, ExAC, ClinVar or 1000 Genomes Project databases. Furthermore, according to the American College of Medical Genetics and Genomics standards and guidelines, the novel genetic variant in ANK1 was predicted to be deleterious.^2^

**Fig.2:**
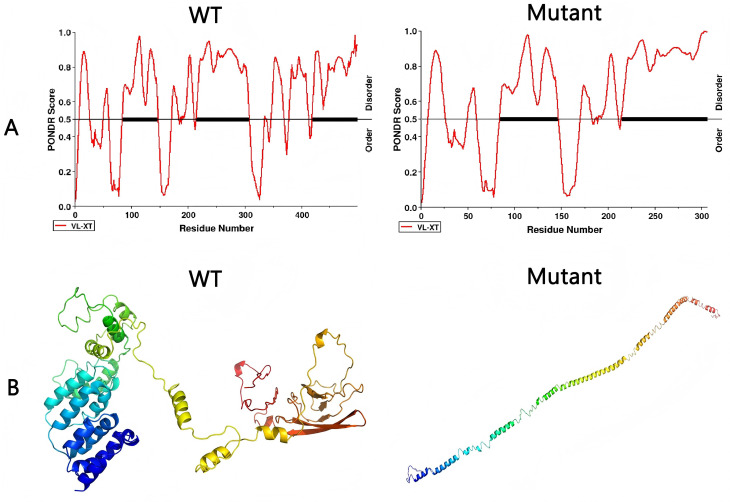
Effect on protein structure and function of frameshift mutation in the mutant or wild-type (WT). A: Prediction of intrinsically disordered residues in human ANK1 by PONDR-FIT; B: 3D structure of ANK1 protein predicted by I-TASSER suite.

To confirm the effect of this genetic variation on protein structure and function, prediction of intrinsically disordered amino acids and homology-modeling analysis were performed. We used the PONDR tool to predict order-disorder of the regulatory domain of ANK1 in the wild-type or frameshift variant.^3^ As shown in Fig.2A there was an obvious ordered region in amino acids 300-350 in the wild type, whereas, this region was completely lost in the mutant. Thus, we considered that the frameshift mutation had an important impact on the structure and stability of the protein. Additionally, 3D models of the target protein were also constructed using the I-TASSER suite (Fig.2B).^4^ The frameshift mutation may cause disintegration of the regulatory domain of ANK1 protein, which cannot form an effective 3D structure, therefore impairing the interactions of the membrane-binding domain.

Thus, the diagnosis of HS was made. Splenectomy was not performed because hemoglobin was normal. The patient received no treatment. After more than one year of follow-up, the levels of hyperbilirubinemia and hemoglobin, as well as the splenomegaly, remained unchanged.

## DISCUSSION

HS, an autosomal-dominant inherited disorder, is characterized clinically by anemia, jaundice, hemolysis, splenomegaly, gallstones and positive family history with a prevalence of one in ten thousand in China.^5^ The 2011 edition of the Guidelines for the Diagnosis and Management of Hereditary Spherocytosis by the British Committee for Standards in Haematology (BCSH) recommends that a definitive diagnosis can be made with a family history of hereditary spherocytosis, typical clinical manifestations of HS (including anemia, jaundice, splenomegaly, etc.), and spherical red blood cells accounting for more than 10% of the total erythrocytes. For suspected atypical cases with an insufficient proportion of spherical red blood cells in the peripheral blood and no positive family history, the diagnosis of this disease requires the application of multiple assays, including erythrocyte membrane protein component analysis and genetic analysis. However, atypical HS is easily misdiagnosed. It is reported that about 10% of HS patients have few or no detectable spherocytes.^6^ Moreover, anemia can be mild or compensated in some patients, accounting for misdiagnosed HS.^7^ In our study, the proband had splenomegaly and hyperbilirubinemia without other clinical manifestations such as anemia or spherocytes. The bone marrow puncture revealed erythroid hyperplasia. All these evidences seemed to support a diagnosis of hemolytic diseases. However, all traditional tests related to hemolysis ruled out the possibility, which presented a confusing picture. The proband suffered from hyperbilirubinemia since early childhood; therefore, NGS was an appropriate method to determine the candidate genes responsible for the inherited and rare disorders because of its efficiency, low cost and time saving.

Finally, a novel mutation (c.5032delA, p.R1678Gfs*12) was identified that could cause a premature termination codon in exon 38 of ANK1, and thereby fail to form an effective 3D protein structure, as predicted by I-TASSER. To date, HS is mainly caused by defects in red blood cell membrane protein encoding genes, including ANK1, SLC4A1, EPB42, SPTB and SPTA1, in which mutations of ANK1 gene account for about half of all HS cases.^8^ ANK1 is a major protein of erythrocytes and anchors transmembrane proteins to the spectrin-actin-based membrane skeleton through spectrin, band three and protein 4.2, which plays an important role in the maintenance of red cell deformability and stability.^9^ ANK1 is a large protein composed of 1880 amino acids, consisting of three structural domains: an N-terminal membrane protein-binding domain that contains the binding site for band 3; a central region containing a spectrin-binding domain; and the least conserved regulatory C-terminal domain that modulates the affinity of the other domains.

A study about the genotype-phenotype association suggests patients that harboring mutated ANK1 on the regulatory domain had the least severe anemia compared with those with mutations located on the membrane-binding domain or spectrin-binding domain.^10^ Consistent with this study, the frameshift variant c.5032delA (R1678Gfs*12), located in the regulatory domain in the C terminus of ANK1, did not affect hemoglobin level in our study. Hence, on account of compensated anemia, splenectomy was not carried although the patient had splenomegaly. Finally, the patient did not receive any treatment.

### Limitations:

One of them was the lack of a molecular investigation of the mutated ankyrin protein. The pathogenicity of the mutation was only verified by in-silico tools (including PONDR and I-TASSER) and that functional experimental assays such as protein expression, subcellular localization, and membrane-binding studies were not performed due to experimental conditions, which is a major limitation of this case report. We might generate animal models and cell lines carrying this genetic defect for research in the future.

## CONCLUSION

In summary, our study is the first to report a novel genetic variation of ANK1 (c.5032delA) contributing to atypical HS without clinical manifestations such as anemia or spherocyte. It should be noted that jaundice and splenomegaly are not merely associated with liver diseases, but also with hemolytic diseases. Meanwhile, our results also demonstrate that WES can provide a powerful approach to diagnose complicated, atypical and heritable diseases. Given the autosomal dominant inheritance pattern of this de novo ANK1 mutation, the proband has a 50% theoretical risk of transmitting the mutation to offspring, despite the de novo nature of the variant. Thus, genetic counseling for family planning is essential, and prenatal or preimplantation genetic diagnosis represents a viable clinical option for the proband.
